# Research on the Properties and Mechanism of Carbon Nanotubes Reinforced Low-Carbon Ecological Cement-Based Materials

**DOI:** 10.3390/ma15186435

**Published:** 2022-09-16

**Authors:** Kai Cui, Jixin Zhang, Jun Chang, Mohanad Muayad Sabri Sabri, Jiandong Huang

**Affiliations:** 1 School of Civil Engineering, Dalian University of Technology, Dalian 116024, China; 2 Peter the Great St. Petersburg Polytechnic University, 195251 St. Petersburg, Russia; 3 School of Civil Engineering, Guangzhou University, Guangzhou 510006, China

**Keywords:** carbon nanotubes, dispersion, mechanical properties, hydration, microstructure

## Abstract

SAC (sulfoaluminate cement) has become a research hotspot as a low-carbon ecological cement. In addition, multi-walled carbon nanotubes have good thermal, mechanical, and electrical properties and can serve as excellent nano-reinforced cement-based fillers. This study explored the dispersion of carbon nanotubes (CNTs) and researched the effect of CNTs on the mechanical properties, hydration process, hydration products, and microstructure of SAC paste, and the mechanism of CNT-enhanced SAC paste was revealed. The results showed that the mechanical properties of SAC paste were significantly improved after the addition of CNTs. When the CNT content was 0.05%, 0.1%, and 0.15%, the compressive strength after 28 d was increased by 13.2%, 18.3%, and 22.5%, respectively; compared with the C0 group (without CNTs), the flexural strength increased by 8.2%, 11.3%, and 14.4%, respectively. The addition of CNTs accelerated the hydration process of SAC paste. Due to the adsorption effect and nucleation effect of CNTs, more hydration products were generated, filling the matrix’s pores and improving its compactness. The mechanism of CNTs enhanced SAC paste was revealed. CNTs and hydration products co-filled the pores, including AFt (ettringite) and AH_3_ (gibbsite). CNTs improve the mechanical properties of SAC paste through filling, bridging, crack bending, deflection, pulling out, and pulling off.

## 1. Introduction

With the development of the cement industry, three series of cement have been formed: Portland cement, aluminate cement, and SAC (sulfoaluminate cement). Portland cement is widely used throughout the construction industry due to its wide range of raw material sources and low cost. With the development of society, science, and technology, higher requirements are put forward for cement-based materials, and cement-based materials are developing in the direction of intelligence and multi-energy [1]. OPC (ordinary Portland cement) can no longer meet the demand, and it is necessary to utilize and expand the use of other cement. Among them, sulfoaluminate cement, as a low-carbon ecological cement, has excellent durability, high early strength, fast setting, and fast hardening [2,3,4,5,6]. Sulfoaluminate cement is widely used in winter construction, emergency repair, and marine and special engineering. In addition, China proposes to reach its carbon peak by 2030 and achieve carbon neutrality by 2060 [7,8]. OPC has many carbon dioxide emissions [9], which does not meet sustainable development goals. Using and expanding the utilization of SAC is in line with the development of the construction industry [10]. Adding some materials can improve compressive strength, and flexural toughness is necessary. There are many studies on methods to improve ordinary Portland cement, such as adding nanomaterials, including CNTs (carbon nanotubes) [11,12,13], GNPs (graphene nanoplates) [14,15,16], GO (graphene oxide) [17,18,19], nano-SiO_2_ [20,21], fibers including steel fibers [22], PVA (polyvinyl alcohol fiber) fibers [23], and whiskers [24]. Micro/nanomaterials enhance micro-mechanical properties, and macro-materials enhance macro-mechanical properties. However, there are few studies on SAC cement-based materials, and the strength of sulfoaluminate cement is prone to shrinkage in the later stage.

CNTs are widely used in cement-based materials; CNTs have a high aspect ratio, Young’s modulus above 1TPa, and tensile strength of 50–200 Gpa [1]. CNTs have excellent mechanical, thermal, and electrical properties and are promising nano-modified cement-based materials [1]; at present, many studies mainly focus on the study of CNT-enhanced OPC. Li et al. studied the effect of CNTs on the OPC hydration product C-S-H, indicating that the number of CNTs can regulate the conversion of C-S-H density and optimize the pore structure [25]. Jr et al. found that a dispersant can delay the hydration of OPC paste [26]. MacLeod et al. showed that CNTs could improve the mechanical properties of OPC slurries, and a heat of hydration experiment showed that the nucleation effect of CNTs could accelerate the hydration process of OPC [27]. Chen et al. found that CNTs could promote the conversion of C-S-H from low density to high density, filling the pores of the matrix [28]. De et al. found that CNTs promoted the hydration of OPC, reduced the setting time, and improved the mechanical properties of OPC materials [29]. Gao et al. studied the effect of CNTs with different diameters on the properties of OPC cement-based materials. They found that CNTs could significantly improve cement-based materials’ compressive and flexural strength, which was attributed to optimizing the pore structure of cement-based materials [30]. Sarvandani et al. studied the effect of COOH-CNTs on cement mortar’s mechanical properties and durability. CNTs can significantly improve cement mortar’s compressive and flexural strength as a bridging factor. The addition of CNTs optimizes the pore structure and enhances the microstructure of cement mortar [31]. Hawreen et al. studied the mechanical properties and shrinkage behavior of CNTs on OPC cement-based materials. The addition of CNTs effectively reduced the shrinkage of cement mortar and improved the mechanical properties of the mortar [32].

Currently, the research on CNT-reinforced cement-based materials focuses on OPC, mainly including macroscopic mechanical properties, microscopic hydration, durability, and engineering applications. There is very little research on CNT-enhanced SAC. We need to explore the mechanical properties, hydration mechanism, and microstructure of CNTs for SAC. This study investigated the dispersion of carbon nanotubes (CNTs) and researched the effects of CNTs on the mechanical properties, hydration process, hydration products, and microstructure of SAC paste, and the mechanism of CNTs enhanced SAC paste was revealed.

## 2. Materials and Methods

### 2.1. Materials

Sulfoaluminate cement was provided by Tangshan Polar Bear Building Materials Co., Ltd. (Tangshan, China). The physical properties and chemical composition are shown in Table 1 and Table 2. Chongqing Sansheng Special Building Materials Co., Ltd. (Chongqing, China) produced superplasticizers with a water-reducing efficiency of 30%. Elkem Materials Co., Ltd. produced silica fume; the composition is shown in Table 2. Shenzhen Nano port Co., Ltd. (Shenzhen, China) provided carbon nanotubes, and the physical parameters of carbon nanotubes are shown in Table 3. According to previous research [6], the mixing ratio of SAC:water: silica fume is 1:0.286:0.1, the superplasticizer dosage is 1–1.2%, and the carbon nanotubes are 0, 0.05%, 0.1%, and 0.15% of the cement mass. The sample labels are C0, C1, C2, and C3.

### 2.2. Preparation and Methods

The dispersion of CNTs was achieved by a combination of mechanical stirring, surfactant, and ultrasonic dispersion. First, different types and dosages of dispersants were weighed and added to a beaker containing 50 mL of deionized water; the solution was stirred with a glass rod for 3 min and then ultrasonically dispersed with an ultrasonic processor for 3 min. After the dispersants were dissolved, the 5 mg of weighed CNTs was introduced into the dispersion, mechanically stirred for 3 min, and ultrasonically treated for 5 min at an output power of 360 W, and finally, the cup was sealed with a plastic film for testing. A UV/Vis spectrophotometer was used to characterize the change in the absorbance of the CNT suspension with the addition of dispersant. After obtaining the optimal amount of dispersant, the dispersion was mixed with SAC and SF and poured into a stirring pot, which was first stirred at low speed for 2 min, then, following the addition of water and superplasticizer, stirred at low speed for 2 min, and finally, stirred at high speed for 3 min. The paste was poured into a 4 × 4 × 16 cm^3^ steel membrane and vibrated on a vibrating table for 2 min, and the mold was placed in a standard curing room (20 °C 95% humidity) for 1 day, after which the mold was removed. Compressive and flexural strength were tested according to the Chinese standard GB/T 17671-1999.

UV-VIS was used to characterize the dispersion of CNTs with a wavelength of 260 nm, and the type of dispersant and the optimal amount of dispersant were determined; FE-SEM (USA, FEI Quanta 450450) was used to observe the morphology of CNTs before and after dispersion and the effect of CNTs on SAC cement. To study the role of the base material, a Tam air C80 microcalorimeter (TA Instruments, Inc., New Castle, DE, USA) was used to measure the exothermic hydration process of SAC with and without CNTs. For XRD (Bruker D8 ADVANCE diffractometer, Billerica, MA, USA) with a test range of 5–80°, the working voltage and working current were 40 kV and 40 mA, respectively. EVA software qualitatively analyzed the composition of hydrated samples with a step size of 0.1 s, and Topas4.2 software quantitatively analyzed the content of hydration products and unhydrated cement clinker with a step size of 0.5 s. TG-DTG analysis (Swiss, Mettler Toledo TGA/DSC1) was used to test the weight loss of hydrated samples at different temperatures. The test temperature was 50 °C to 800 °C, the heating rate was 10 °C/min, and the protective gas was nitrogen.

## 3. Results and Analysis

### 3.1. Dispersion of CNTs

Based on the Lambert–Beer law, the dispersion effect of CNTs in the solution was characterized. The absorbance *A* was calculated by Equation (1) [33].

(1) $$A=\mathrm{lg}(\frac{I_{r}}{I_{s}})=ECL$$

where *E* is a constant, i.e., the absorption coefficient of the light-absorbing substance. *C* is solution concentration, and *L* is the optical path length. It can be seen that when the optical path length *L* is constant, the absorbance *A* is proportional to the solution concentration C. $I_{r}$ indicates the intensity of incident monochromatic light, and $I_{s}$ indicates the transmitted light intensity.

**Figure 1:**
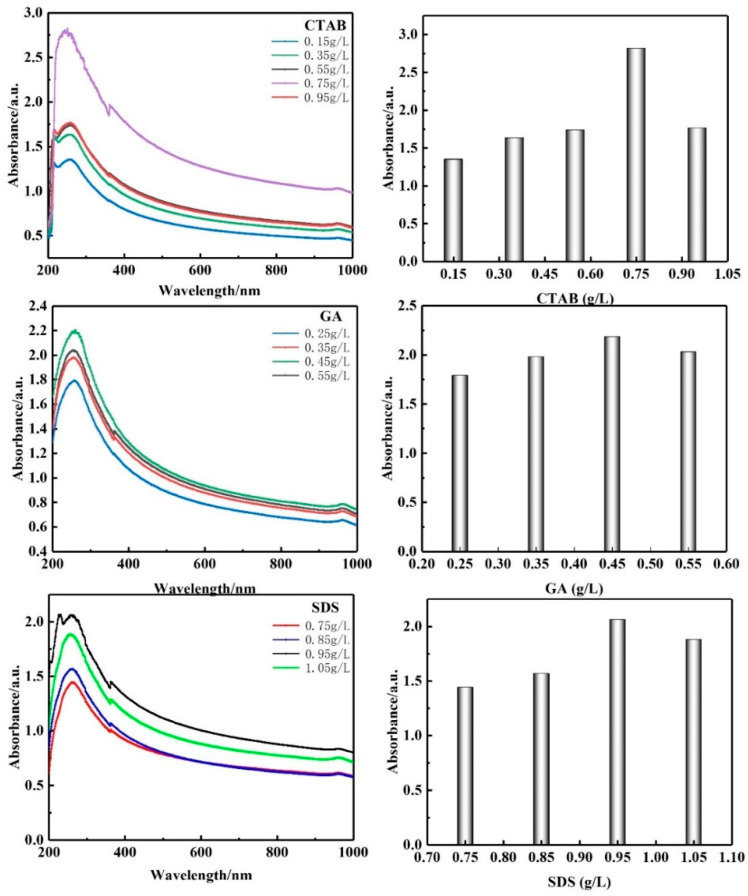
UV spectra of CNT dispersions and absorbance of CNT dispersions.

**Figure 2:**
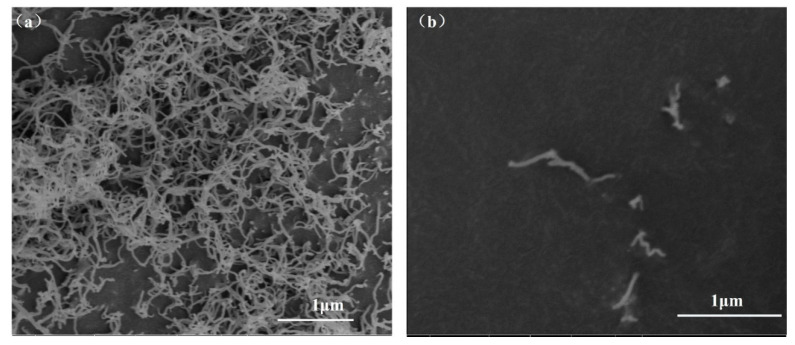
SEM of CNTs: (**a**) before dispersion; (**b**) after dispersion.

According to previous studies, CTAB (cetyl trimethyl ammonium bromide), GA (Gum Arabic), and SDS (sodium dodecyl sulfate) were used as dispersants. The concentration range of CTAB was 0.15 g/L~0.95 g/L, the concentration range of GA was 0.25 g/L–0.55 g/L, and the concentration range of SDS was 0.75 g/L–1.05 g/L. UV-VIS was used to test the absorbance of CNTs with different dispersant concentrations, as shown in Figure 1. With the change in CTAB, GA, and SDS contents, the absorption peak at 260 nm and the absorbance of dispersion liquid showed an increasing trend. When the CTAB concentration was 0.75 g/L, the GA concentration was 0.45 g/L, and the SDS concentration was 0.95 g/L, the absorbance of CNTs reached maximum values of 2.819, 2.185, and 2.062, respectively. According to the Beer–Lambert law, CNTs have a better dispersion effect. Figure 2 shows the morphology of CNTs before and after dispersion. It can be seen that the state of CNTs before and after dispersion is significantly different: CNTs without dispersion form larger agglomerates, and CNTs are intertwined with each other and cannot uniformly disperse. The states are separated; single dispersed CNTs can be seen uniformly, and dispersed CNTs are uniformly distributed in the matrix to form a wide spatially enhanced network.

### 3.2. Macro-Performance

**Figure 3:**
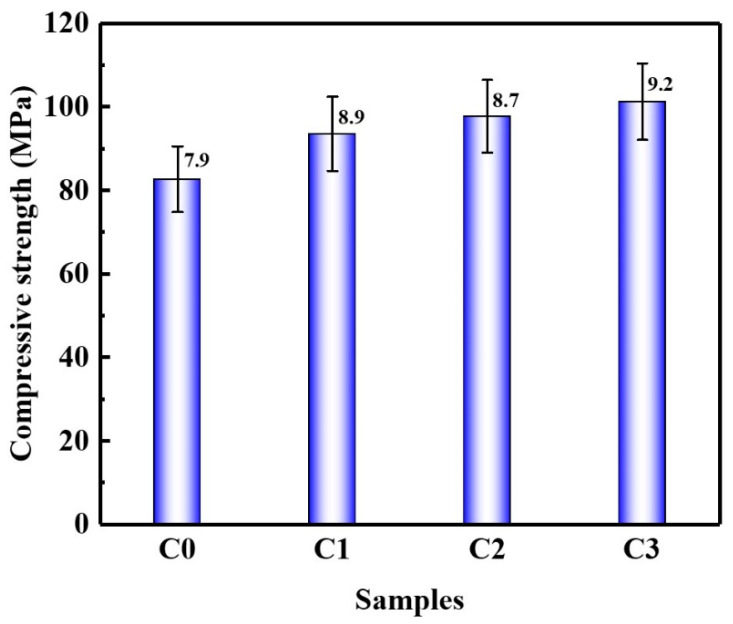
Compressive strength of SAC paste.

**Figure 4:**
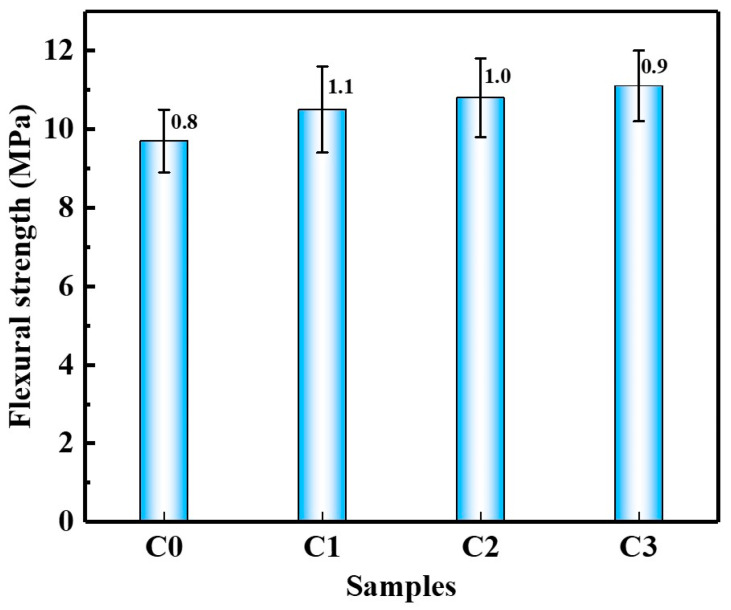
Flexural strength of SAC paste.

Figure 3 shows the compressive strength of SAC pastes with and without CNTs after curing for 28 d. It can be seen that after the addition of CNTs, the compressive strength of SAC paste was improved, and the compressive strength increased with the increase in CNT content. The compressive strength of the C1, C2, and C3 groups was increased by 13.2%, 18.3%, and 22.5%, respectively, compared with the C0 group. Figure 4 shows the flexural strength of SAC pastes with and without CNTs after curing for 28 d. The changing trend of flexural strength of SAC paste was similar to that of compressive strength, and the flexural strength increased with the increase in CNT content. The flexural strengths of the C1, C2, and C3 groups were increased by 8.2%, 11.3%, and 14.4%, respectively, compared with the C0 group. CNTs improved the mechanical properties of SAC slurries, which can be attributed to the fact that CNTs can fill the pores of the matrix and improve the microstructure [27].

### 3.3. Composition and Content of Hydration Products

#### 3.3.1. Heat of Hydration

**Figure 5:**
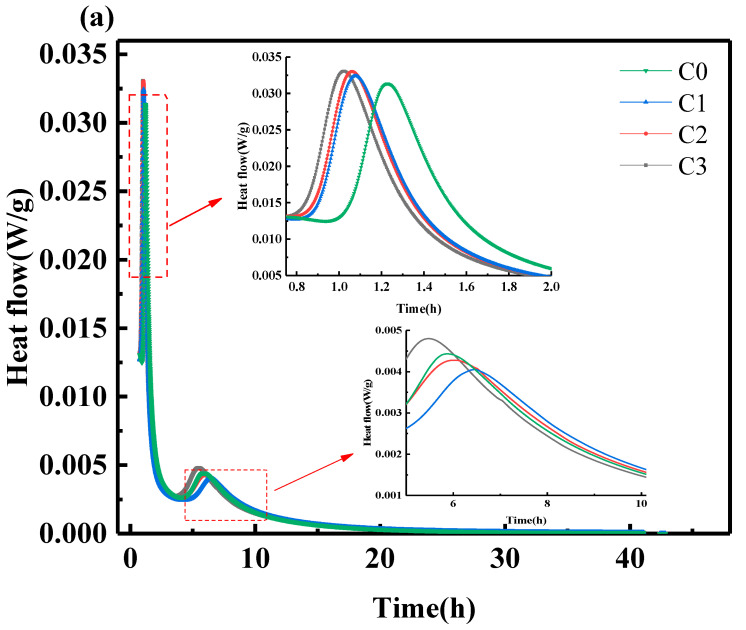

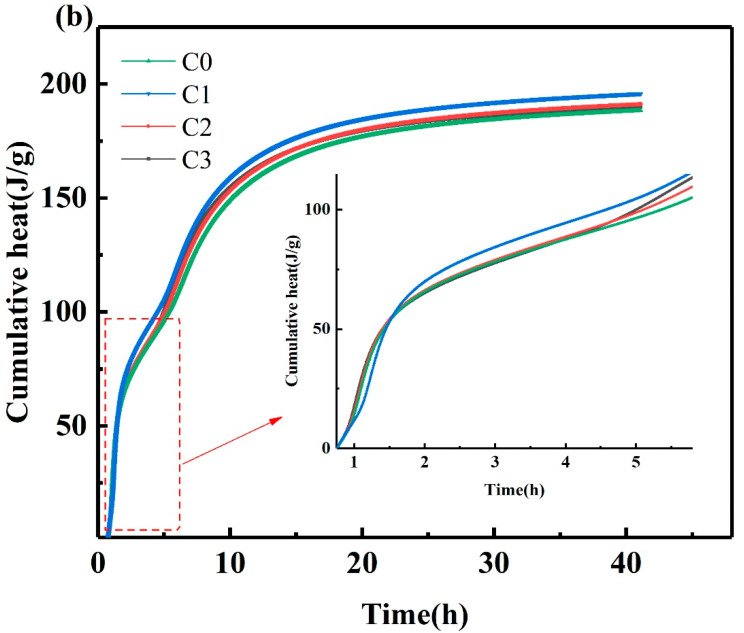
Hydration heat: (**a**) hydration flow; (**b**) cumulative heat.

The heat of hydration is an essential physical parameter to evaluate the hydration exotherm of a sample. According to previous studies [7], the hydration of SAC mainly consists of five stages: the dissolution period, induction period, acceleration period, deceleration period, and stabilization period. It can be seen in Figure 5 that the hydration exotherm of SAC paste was mainly concentrated in the first 24 h. This study tested the hydration exotherm of SAC paste for 40 h. When the cement meets water, the clinker minerals begin to dissolve. This is the dissolution period; then, hydration enters the induction period, which is very short, and hydration enters the acceleration period. During the acceleration period, two exothermic hydration peaks appear. One corresponds to the consumption of calcium sulfoaluminate and the formation of AFt (ettringite). As the hydration reaction proceeds, the generated hydration product AFt covers the surface of the clinker, and the heat release rate decreases. The film ruptures with the increase in osmotic pressure inside and outside the clinker, and AFt is formed, corresponding to the appearance of the second exothermic peak [7].

As can be seen in Figure 5a, the first exothermic peaks of C0, C1, C2, and C3 appeared at 73.45 min, 64.67 min, 63.79 min, and 61.16 min, respectively. Compared with C0, the exothermic peaks of C1, C2, and C3 were 8.78 min, 9.66 min, and 12.29 min earlier, respectively. It is shown that the addition of CNTs accelerated the hydration exothermic rate of SAC due to the adsorption effect of nanomaterials, which promoted the dissolution of clinker, and the nucleation effect of nanomaterials, which promoted the formation of hydration products [26]. The second exothermic peaks of C0, C1, C2, and C3 samples appeared at 5.81 h, 6.41 h, 5.96 h, and 5.42 h, respectively.

Figure 5b shows the cumulative heat release of SAC paste for 40 h. When the first exothermic peak appeared in C0, the cumulative heat release rates corresponding to C0, C1, C2, and C3 were 37.97 J/g, 28.93 J/g, 39.29 J/g, and 40.43 J/g, respectively. When the second exothermic peak appeared in C0, the cumulative exothermic heat rates of C0, C1, C2, and C3 were 105.25 J/g, 116.03 J/g, 109.8 J/g, and 113.7 J/g, respectively. After adding CNTs, the cumulative heat release of C1, C2, and C3 samples was greater than that of C0, indicating that CNTs accelerated the hydration heat release rate of SAC and promoted the formation of hydration products. After 40 h of hydration, the cumulative heat release rates of C0, C1, C2, and C3 were 188.03 J/g, 195.56 J/g, 190.86 J/g, and 189.67 J/g. When the CNTs content was 0.05%, the cumulative heat release was the largest.

#### 3.3.2. XRD and TG Analysis

**Figure 6:**
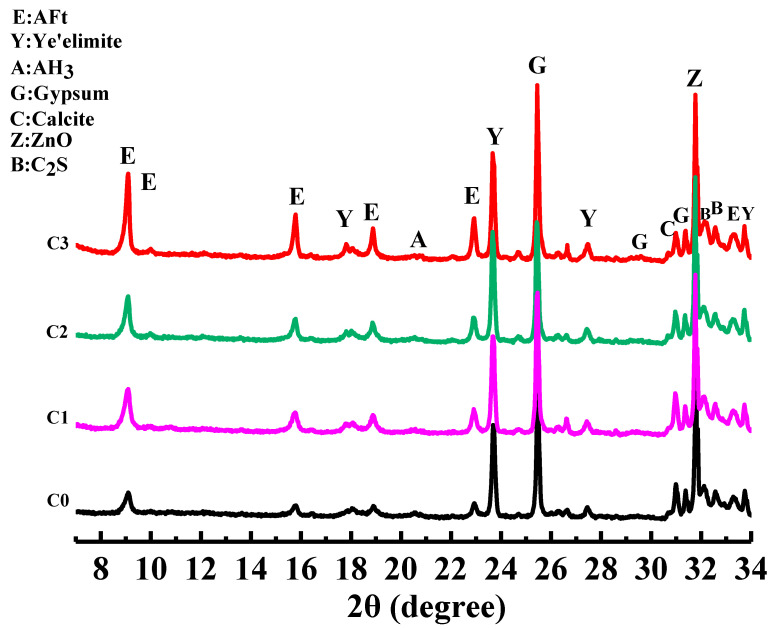
XRD patterns of SAC paste.

Figure 6 shows the XRD pattern of SAC paste for 28 days. The figure shows the diffraction peaks of the hydration products AFt and AH_3_ (gibbsite) and the diffraction peaks of the unhydrated clinker C_4_A_3_Š (ye’elimite), C_2_S, and gypsum. The AFt peak intensity of the sample after adding CNTs was higher than that of the C0 group. The peak intensity of C_4_A_3_Š was lower than that of the C0 group. No new diffraction peaks were formed, indicating that SAC hydration was promoted after the addition of CNTs. Still, no chemical reaction occurred, which is consistent with the notion that CNTs promote OPC hydration; the adsorption effect of CNTs promotes the hydration of SAC. The nucleation effect of CNTs provides reaction sites for forming hydration products.

**Figure 7:**
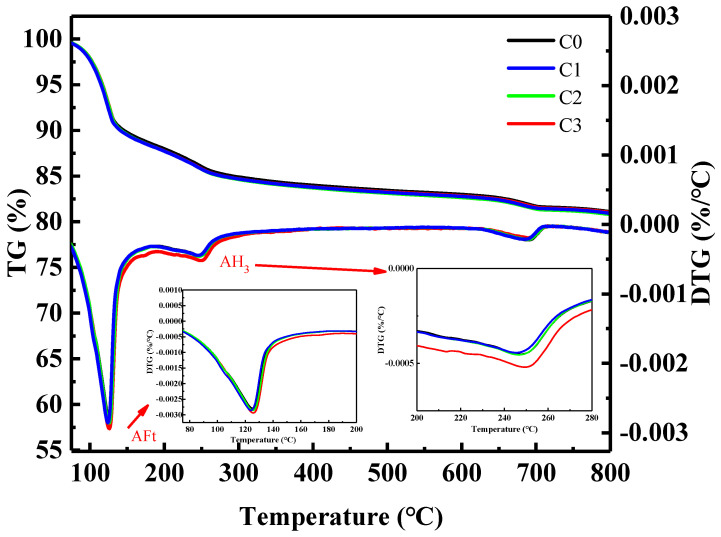
TG-DTG curves of SAC paste.

As shown in Figure 7, there are four weight loss peaks. The first peak appeared at 100–150 °C, corresponding to the decomposition of AFt. The second peak occurred at 150–200 °C, corresponding to the decomposition of gypsum. The third peak appeared at 210–270 °C, corresponding to the decomposition of AH_3_. The fourth peak occurred at 650–800 °C, corresponding to the decomposition of calcium carbonate. There is no weight loss peak at 450–510 °C, indicating no generation of CH, which is consistent with the analysis results of XRD. It can be seen in Figure 7 that the weight loss peaks of AFt and AH_3_ in the C1, C2, and C3 groups are greater than those in the C0 group, indicating that the addition of CNTs promotes the hydration of SAC and generates more hydration products.

#### 3.3.3. Hydration Degree

Since XRD cannot directly detect the phase content, the Rietveld method (QRXD) was used to quantitatively calculate the hydration product content and unhydrated clinker content of samples hydrated for 28 days. The R_WP_ value of the fitted curve is lower than 15%, indicating that the fitting effect is good. After 28 days of curing, the AFt contents of the samples in each group were 13.79%, 14.24%, 14.75%, and 15.87%, respectively. It was observed that the content of AFt increased with the content of CNTs; the Amor (amorphous) contents of the samples after curing for 28 days were 37.49%, 38.16%, 38.76%, and 39.98%, respectively. Because Amor contains AH_3_, the changing trend of Amor content is similar to that of AFt, and AH_3_ is also one of the main hydration products of SAC. AH_3_ is microcrystalline or amorphous and cannot be accurately quantified by QXRD.

**Figure 8:**
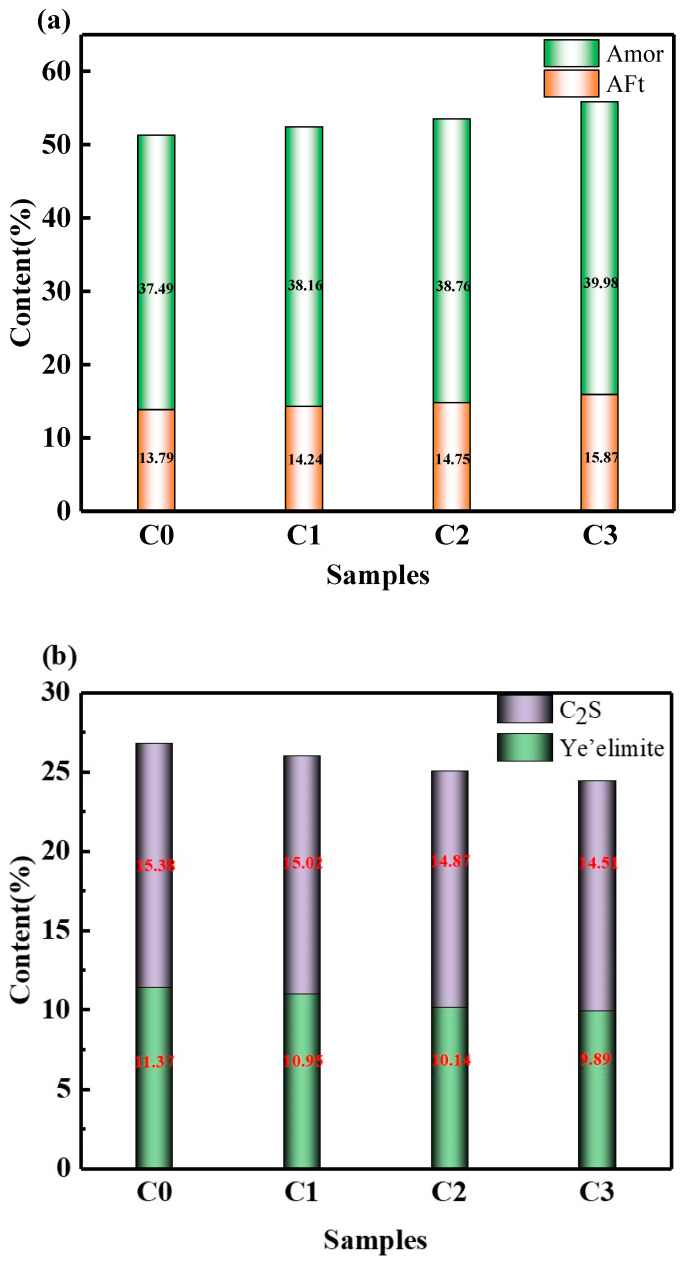
Contents of hydration products and unhydrated clinker (**a**,**b**).

The changing trend of C_4_A_3_Š is shown in Figure 8; the content of C_4_A_3_Š after curing for 28 days was 11.37%, 10.95%, 10.14%, and 9.89%, respectively. The changing trend of the content of C_4_A_3_Š was opposite to that of AFt because the hydration reaction of C_4_A_3_Š generates AFt. Consistent with the results of qualitative XRD analysis, the changing trend of C_2_S content is shown in Figure 8; after curing for 28 days, the C_2_S content was 15.38%, 15.02%, 14.87%, and 14.51%. The contents of C_4_A_3_Š and C_2_S decreased with the increase in CNTs, indicating that CNTs promoted the hydration process of SAC paste, and the QXRD results are in good agreement with the TG and hydration thermal analysis results.

According to Equation (2) [7], the hydration degree can be calculated:

(2) $$\alpha_{t}=\frac{m_{0}-m_{t}}{m_{0}}\times 100\%$$

where $\alpha_{t}$ is the hydration degree, and the values of $m_{0}$ and $m_{t}$ in this paper are the results of QXRD.

**Figure 9:**
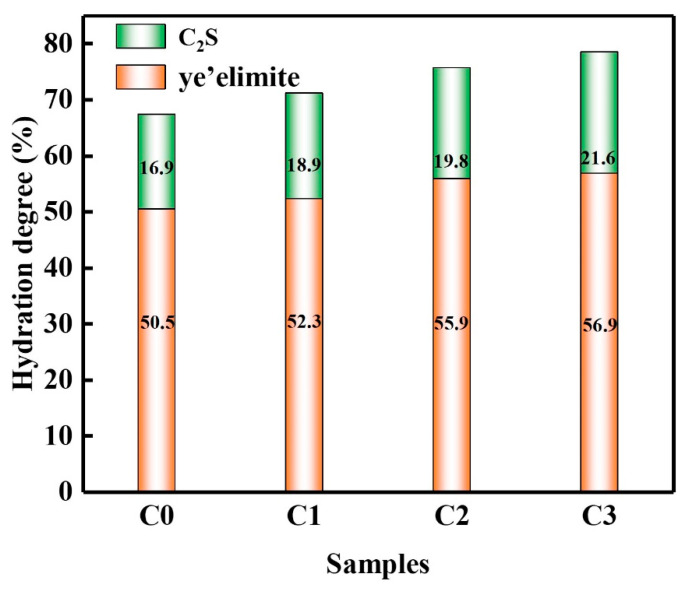
Hydration degree of SAC paste.

The hydration degrees of the samples are shown in Figure 9a, and the hydration degrees of the hydrated sample C_4_A_3_Š were 50.5%, 52.3%, 55.9%, and 56.9%, respectively. From the trend of the hydration degree of C_4_A_3_Š, CNTs accelerated the early hydration of SAC paste. The hydration degree of C_2_S was similar to that of C_4_A_3_Š, as shown in Figure 9b. The hydration degree of C_2_S was 16.9%, 18.9%, 19.8%, and 21.6%, respectively. According to the degree of hydration of C_4_A_3_Š and C_2_S, it can be concluded that CNTs promote the hydration process of SAC paste.

### 3.4. SEM Observation

**Figure 10:**
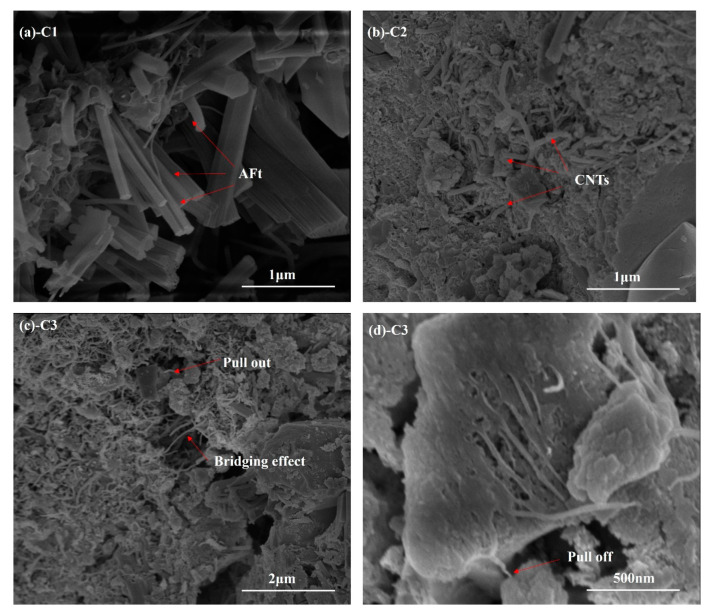
SEM observation of SAC paste.

Figure 10 shows the morphology of CNTs in SAC cement paste. It can be seen that CNTs are widely distributed in the matrix and form a reinforcing network, as shown in Figure 10a,b, which is conducive to the enhancement of the mechanical properties of the SAC matrix by CNTs. The mechanism of action of CNTs on the SAC matrix mainly includes the following aspects. CNTs bridge the nano- and micro-sized cracks in the matrix. After the micro-scale cracks are generated, they will further develop and expand under the stress state. When the development direction of the cracks encounters the CNTs, the bridging effect of CNTs effectively prevents the cracks. As the crack continues to develop, when the SAC matrix is subjected to external force, the CNTs inside the matrix will be pulled, as shown in Figure 10c.

When the pulling force is greater than the bonding force between the CNTs and the matrix, the CNTs will be pulled out or off the matrix, as shown in Figure 10d. The failure state of CNTs is related to the critical length. When the actual length of CNTs is less than or equal to the critical length, the failure state of CNTs in the matrix is mainly based on pull-out failure. When the actual length is greater than the critical length, the failure state of CNTs in the matrix is primarily based on pull-off failure. Pulling CNTs out or off must overcome the adhesion and friction between CNTs and the matrix; it is an essential energy-consuming process. In addition, cracks extend and develop in the matrix. After encountering CNTs, they extended along the direction of CNTs, and the cracks bent and deflected, significantly increasing the cracks’ development path in the matrix. The nucleation of CNTs promoted the hydration of SAC. It generated more hydration products, including AFt and AH_3_, and the developed hydration products and CNTs filled the SAC matrix pores, improving the matrix compactness and the CNT and matrix. The adhesive force is beneficial in enhancing the mechanical properties of SAC.

## 4. Conclusions

This study investigated the dispersion of carbon nanotubes (CNTs) and researched the effect of CNTs on the mechanical properties, hydration process, hydration products, and microstructure of SAC paste, and the mechanism of CNTs-enhanced SAC paste was revealed. The following conclusions were drawn.

1. The addition of CNTs accelerated the hydration process of SAC paste. Due to the adsorption effect and nucleation effect of CNTs, more hydration products were generated, filling the matrix’s pores and improving its compactness.
2. The mechanical properties of SAC paste were significantly improved after the addition of CNTs. When the CNT content was 0.05%, 0.1%, and 0.15%, the compressive strength after 28 d was increased by 13.2%, 18.3%, and 22.5%, respectively, compared with the C0 group, and the flexural strength increased by 8.2%, 11.3%, and 14.4%, respectively.
3. The mechanism of CNTs enhanced SAC paste was revealed. CNTs and hydration products, including AFt and AH_3,_ co-filled the pores. CNTs improve the mechanical properties of SAC paste by filling, bridging, crack bending, deflection, pulling out, and pulling off.

## Figures and Tables

**Table 1:** Physical properties of SAC.

Setting Time/Min		Density (g/cm^3^)	Flexural Strength/MPa		Compressive Strength/MPa	
Initial Setting	Final Setting		3 d	28 d	3 d	28 d
27	43	3.1	6.2	7.1	45.1	54.3

**Table 2:** Chemical composition of SAC and SF (wt%).

Type	Fe_2_O_3_	Al_2_O_3_	SO_3_	MgO	SiO_2_	CaO	Density (g/cm^3^)	Loss on Ignition
SAC	2.13	17.62	11.95	2.43	18.16	46.31	3.1	≤1.4
SF	0.64	0.71	0.15	0.13	94.36	0.11	2.0	≤3.9

**Table 3:** Characteristics of CNTs.

Type	Outer Diameter/nm	Surface Area /m^2^·g^−1^	Length/(μm)	Grayscale	Purity/%
L-MWCNTs	20–40	80–140	5–15	<3 wt%	>97
